# Supplementary figures and images for: Complete Chloroplast Genomes of Fagus sylvatica L. Reveal Sequence Conservation in the Inverted Repeat and the Presence of Allelic Variation in NUPTs
Source: Genes (Basel). 2021 Aug 29;12(9):1357. doi: 10.3390/genes12091357 (PMC8468245; doi:10.3390/genes12091357)

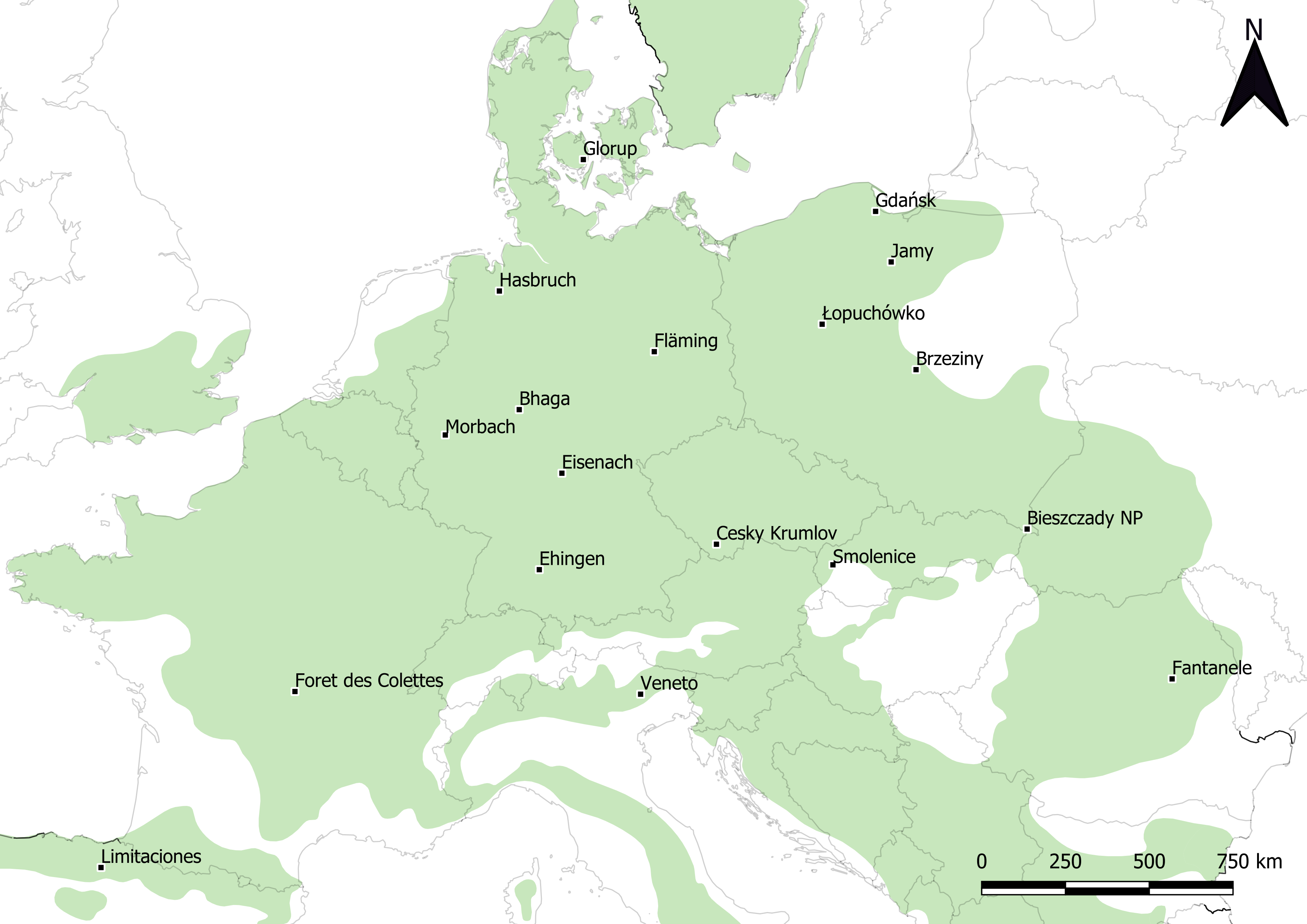

Supplement: Supplementary file 1 [file genes-12-01357-s001.zip › genes-1253124-supplementary.png]
